# The right time to measure anti-Xa activity in critical illness: pharmacokinetics of therapeutic dose nadroparin

**DOI:** 10.1016/j.rpth.2023.100185

**Published:** 2023-05-20

**Authors:** Jelmer G. Sytema, Bert G. Loef, Harriët M. Loovers, Marijn Boer, Daniël J. Touw, Marinus van Hulst

**Affiliations:** 1 Department of Clinical Pharmacy, Martini Hospital, Groningen, the Netherlands; 2 Department of Clinical Pharmacy, Hospital Nij Smellinghe, Drachten, the Netherlands; 3 Intensive Care Unit, Martini Hospital, Groningen, the Netherlands; 4 Department of Clinical Chemistry, Certe, Groningen, The Netherlands; 5 Department of Clinical Pharmacy and Pharmacology, University of Groningen, University Medical Centre Groningen, Groningen, the Netherlands; 6 Department of Health Sciences, University of Groningen, University Medical Center Groningen, Groningen, the Netherlands

**Keywords:** anti-Xa, critical illness, LMWH, low-molecular-weight heparin, nadroparin, pharmacokinetics

## Abstract

**Background:**

Peak anti-Xa activity of low-molecular-weight heparin nadroparin is measured 3 to 5 hours after subcutaneous injection. In critically ill patients, physiological changes and medical therapies may result in peak activities before or after this interval, possibly impacting dosing.

**Objectives:**

The primary objective was to determine the percentage of critically ill patients with adequately estimated peak activities drawn 3 to 5 hours after subcutaneous administration of a therapeutic dose of nadroparin. Adequate was defined as a peak activity of ≥80% of the actual peak anti-Xa activity. If ≥80% of patients had adequately estimated peak activities in the 3- to 5-hour interval, measurement in this interval was regarded as acceptable. The secondary objective was to determine the pharmacokinetic profile of nadroparin.

**Methods:**

In this single-center, prospective study, we evaluated anti-Xa activities in patients admitted to a general intensive care unit. After ≥4 equal doses of nadroparin, anti-Xa activity was measured according to a 12- to 24-hour sampling scheme.

**Results:**

In 25 patients, anti-Xa activities drawn between 3 and 5 hours after administration ranged 80% to 100% of the actual peak activity. Compared to the threshold level of an adequate estimation in at least 20 patients (≥80%), measuring anti-Xa activities in the 3- to 5-hour interval is an acceptable method (1-tailed binomial test; *P* < .02). We found a large interindividual variability for nadroparin exposure (mean ± SD area-under-the-curve_0-12h_, 10.3 ± 4.8 IU·h/mL) and delayed elimination (t_1/2_ range, 4.0-120.9 hours) despite adequate renal function.

**Conclusion:**

In critically ill patients, measuring anti-Xa activity in a 3- to 5-hour interval after subcutaneous injection of therapeutic nadroparin is an acceptable method to estimate the actual peak anti-Xa activity.

## Essentials

• In critically ill patients, measuring peak anti-Xa levels 3 to 5 hours after nadroparin injection may not be valid.
• After ≥4 doses of nadroparin, anti-Xa was measured using a 12- to 24-hour sampling scheme.
• Measuring anti-Xa levels 3 to 5 hours after injection is acceptable to estimate the peak anti-Xa levels.
• Ultralong elimination half-lives up to 121 hours and 6-fold variation in exposure were found.

## 1 Introduction

Low-molecular-weight heparins (LMWHs) in a therapeutic dose are prescribed to critically ill patients for the treatment of venous thromboembolism and the prevention of stroke in atrial fibrillation. Although the use of anti-Xa activity for monitoring the antithrombotic effect of LMWHs is debated, measuring anti-Xa activity is generally suggested in patients with less predictable pharmacokinetics (PKs), such as in renal insufficiency, obesity, and during pregnancy [[1], [2], [3], [4], [5], [6], [7]]. Target ranges of anti-Xa peak activity (C_max_) in the American College of Chest Physicians guideline “Parenteral Anticoagulants” were derived from anti-Xa activities found in limited PK studies [2].

The time to determine the C_max_, hence the t_max_, was generally taken as 4 hours after a subcutaneous dose of a LMWH despite different LMWHs varying in their PKs, and relevant variation in t_max_ can be expected within and between patients [2,8]. Although few clinical and PK studies have been carried out with nadroparin, nadroparin was assumed to have similar target ranges of anti-Xa peak activities and t_max_ as enoxaparin [2]. In contrast, however, the t_max_ for the peak anti-Xa activity of nadroparin may vary from less than 3 to 9 hours [[9], [10], [11], [12]]. In practice and as recommended by the Dutch Society for Internal Medicine (Nederlandse Internisten Vereniging [NIV]), peak anti-Xa activity is measured 3 to 5 hours after subcutaneous administration of an LMWH [5]. Peak anti-Xa activity is expected to range from 0.6 to 1.0 IU/mL for a twice-daily therapeutic dose of an LMWH and from 1.0 to 2.0 IU/mL for a once-daily dose.

Until now, PK studies with therapeutic nadroparin have not been carried out in critically ill patients [10]. Notably, in critically ill patients, various physiological changes and use of vasopressive drugs may lead to altered PKs affecting absorption, distribution, and elimination of nadroparin [12,13]. These alterations in the PK profile may introduce relevant time shifts to reach the peak anti-Xa activity [10]. Hence, the peak anti-Xa activity measured in an interval 3 to 5 hours after subcutaneous nadroparin administration may not reflect the actual peak anti-Xa activity. Because peak anti-Xa activities can be used to adjust the nadroparin dosage, these time shifts to reach the peak activity may have an impact on the nadroparin dosage that is prescribed [5].

The primary objective of this study was to examine the incidence of underestimated peak anti-Xa activities in general intensive care patients by measuring anti-Xa activities 3 to 5 hours after nadroparin administration. Anti-Xa activities <80% of the actual anti-Xa peak level were considered to be underestimated [14,15]. The secondary objective was to describe the PK profile of a subcutaneous therapeutic nadroparin dose in these patients.

## 2 Methods

### 2.1 Study population

Patients admitted to the intensive care unit (ICU) of the Martini Hospital (the Netherlands) were assessed for eligibility from August 2020 to June 2021. The criteria for inclusion were the following: (1) admission to the ICU and treated with nadroparin (Fraxiparine, 9500 IE/mL concentration; Mylan Ltd, Amstelveen, The Netherlands) in a therapeutic dose (once daily or twice daily) and (2) age ≥18 years. Patients were excluded from study participation if they met any of the following criteria: (1) pregnancy; (2) requiring hemodialysis (HD) or continuous veno-venous hemo(dia)filtration; (3) treatment with a direct oral anticoagulant, unfractionated heparin, another LMWH, or a glycoprotein IIb/IIIa receptor antagonist 96 to 0 hours prior to the first blood sample drawn or during blood sampling; (4) treatment with Fraxiparine forte (nadroparin 19,000 IE/mL concentration) on the day prior to the study day; and (5) participation in another study.

The study protocol and consent form were approved by the Medical Ethical Committee (Regionale Toetsingscommissie Patiëntgebonden Onderzoek [RTPO]), and all subjects or their legal representatives provided written informed consent before actual inclusion. The study was registered at the Netherlands Trial Register (www.trialregister.nl, VARIAXA, Trial NL8205).

## 2.2 Study protocol

This was a single-center, prospective observational PK study.

Patients received the standard dose of nadroparin based on their weight and renal function (local protocol, Table 1). Nadroparin was administered subcutaneously into the thigh or abdomen, and the exact time and site of injection was recorded.

**Table 1:** Nadroparin dosage based on weight and renal function (local guideline).

Bodyweight (kg)	Dosage		
	GFR ≥ 50 mL/min	GFR ≥ 30-49 mL/min	GFR < 30 mL/min
<50	3800 IU every 12 h	2850 IU every 12 h	3800 IU every 24 h
50-70	5700 IU every 12 h	3800 IU every 12 h	5700 IU every 24 h
70-90	7600 IU every 12 h	5700 IU every 12 h	7600 IU every 24 h
90-110	9500 IU every 12 h	7600 IU every 12 h	9500 IU every 24 h

GFR, glomerular filtration rate.

Data on baseline characteristics were collected for each patient, including age, weight on study day, C-reactive protein, serum creatinine, vasopressor use, invasive mechanical ventilation, fluid balance, the Sequential Organ Failure Assessment score, and Acute Physiology and Chronic Health Evaluation. Twenty-four–hour urine sample collection was performed on the study day for measurement of creatinine clearance (CCr).

### 2.3 Sampling and laboratory methods

Blood was drawn from an indwelling arterial catheter after at least 4 equal doses of nadroparin (steady state). Anti-Xa activity was determined just before the administration of nadroparin (t = 0); 1, 2, 2½, 3, 3½, 4, 4½, 5, 5½, 6, 7, 9, and 12 hours after nadroparin administration; and just before the next nadroparin injection in case of once-daily treatment.

Blood samples were collected in 3.2% buffered sodium citrate–containing tubes and then centrifuged at 2500 × *g* for 15 minutes at 20 °C. The obtained plasma samples were aliquoted in 2 plastic tubes and both aliquots were analyzed within 1 hour (duplicate measurement). The samples were subsequently stored at −80 °C. Antithrombin activity was measured from frozen samples in the first or second sample taken. Interference by triglycerides, icterus, hemolysis, or residual thrombocytes on anti-Xa activity measurements was excluded.

Anti-Xa activity was measured using a chromogenic anti-Xa assay (INNOVANCE Heparin, Siemens). Antithrombin activity was measured using a chromogenic assay (INNOVANCE Antithrombin, Siemens). Assays were performed using the CS2500 coagulation analyzer (Siemens Healthineers). Measurements were performed according to the manufacturer’s instructions and using 1 identical lot number of reagent.

The anti-Xa standard calibration curve of nadroparin ranged from 0.10 to 1.50 IU/mL. The reported limit of quantification was below the lower limit of the assay range. The limit of blank (LOB) was <0.15 IU/mL. Samples with activities >1.50 IU/mL were diluted 1:1 with a commercial pool of normal citrated human plasma (Visucon-F Frozen Unassayed Normal Control Plasma, Affinity Biologicals) and reanalyzed.

The interassay precision (coefficient of variation) of the lower (LQC; 0.4 IU/mL) and higher quality control (HQC; 1.0 IU/mL) samples (INNOVANCE Heparin LMW Control 1 and 2, Siemens) was determined at 2.8% and 3.1%, respectively.

### 2.4 PK analysis

For all patients, the PK parameters of nadroparin were derived from the pharmacodynamics of the anti-factor Xa activity. The mean of the measured anti-Xa activities at each sample point was used for further data analysis. If applicable, the first anti-Xa activity below the LOB in the elimination phase or the last anti-Xa activity below the LOB in the absorption phase was considered to be 50% of this LOB. Any later time points with results below the LOB in the elimination phase or earlier points below the LOB in the absorption phase were treated as a missing value.

The noncompartmental PK parameters of nadroparin were obtained from the anti-Xa activity vs time data. The measured anti-Xa peak activity (C_max_) was determined at the corresponding time t_max_. C_min_ was the trough anti-Xa activity before the next dose. The area under the anti-Xa activity vs time curve (AUC) was determined by using the trapezoidal rule; AUC_(0-12h)_ in a 12-hour dosing interval and AUC_(0-24h)_ in a 24-hour dosing interval (GraphPad, version 9.0.2, GraphPad software Inc). The apparent absorption rate constant (k_a_) and the apparent elimination rate constant (k_el_) were obtained by linear regression analysis, calculated as the slope of the rising part and the negative of the slope of the terminal part of the semilogarithmic anti-Xa versus time curve (GraphPad), respectively.

### 2.5 Data and statistical analysis

An anti-Xa activity in the 3- to 5-hour interval was defined as an underestimated peak anti-Xa activity if the activity was <80% of the actual peak anti-Xa activity (C_max_). If the percentage of patients with adequately estimated peak anti-Xa activities in the 3- to 5-hour interval was ≥80%, anti-Xa activity measurement in this interval was regarded as an acceptable method to estimate the peak anti-Xa activity.

The 1-sample proportion test (binomial test on a single proportion) was used to determine whether the real population proportion of adequate peak activities (P1) was significantly different from the requirement that measuring anti-Xa in the 3- to 5-hour time frame generates an adequately estimated peak activity in at least 80% of patients (proportion P0). We calculated that 25 patients would be needed to detect a difference between both proportions P1 and P0, with a significance activity alpha of 5% (1-sided test).

Continuous variables are expressed as mean ± SD for normally distributed data and with median (range) for nonparametric distributions. Categorical data are expressed as counts (percentages).

Data were analyzed using IBM SPSS Statistics 20.0.01 (IBM Corp). Graphs were drawn in GraphPad Prism (GraphPad, version 9.0.2, GraphPad software Inc).

## 3 Results

**Figure 1:**
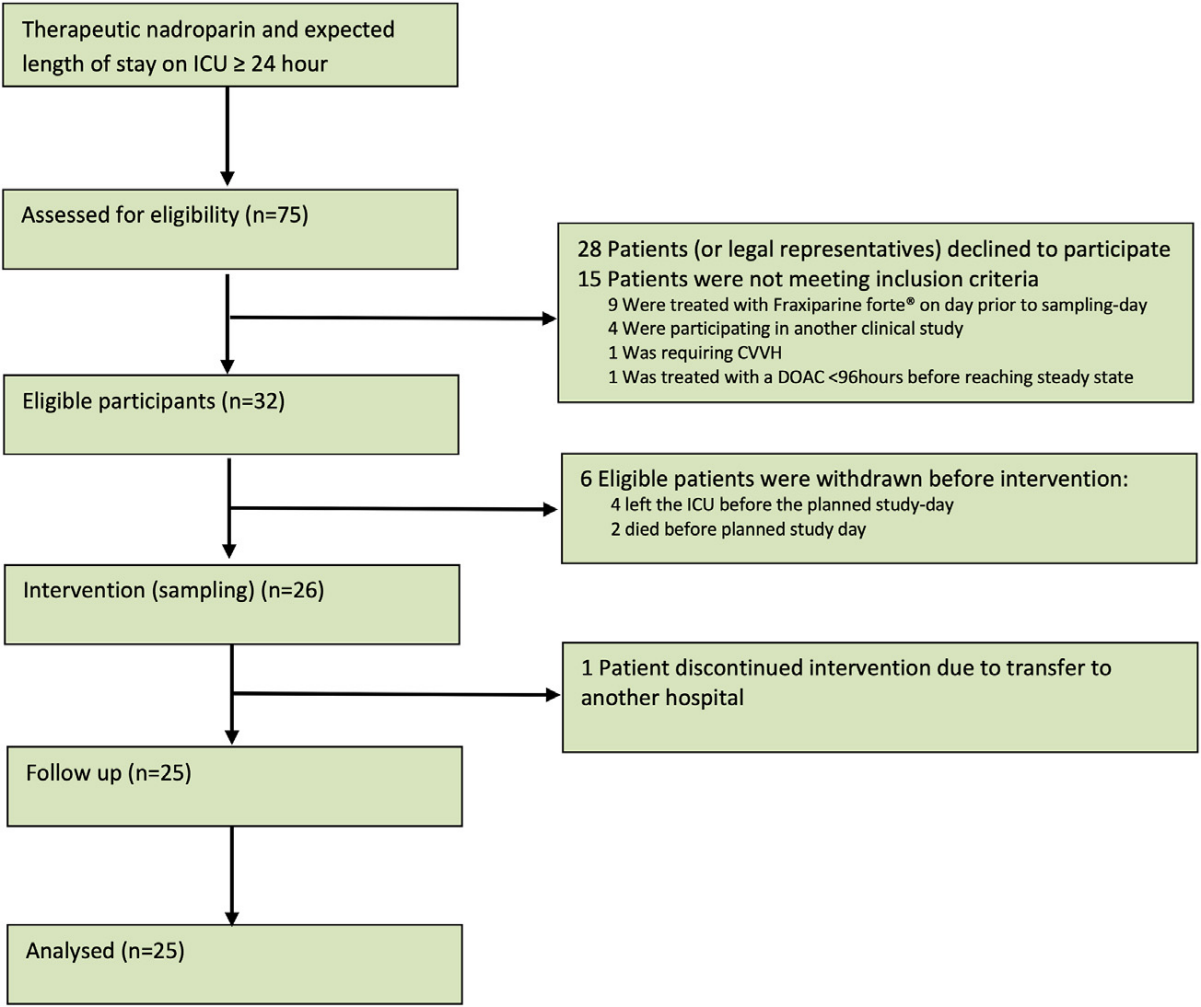
Consort diagram. In total, 28 patients (or legal representatives) declined to participate; due to legal requirements, no data are available on these patients. CVVH, continuous veno-venous hemo(dia)filtration; DOAC, direct oral anticoagulant; ICU, intensive care unit.

In the study period, 75 patients were assessed for eligibility, of which 28 patients (or legal representatives) declined to participate. In total, 25 patients were evaluated in this study (see flowchart in Figure 1). Demographic and clinical characteristics of the study participants are shown in Table 2.

**Table 2:** Demographic and clinical data.

Characteristics	Results (n = 25)
Age (y)	66 ± 9 (50 to 79)
Sex (F/M), n (%)	5/20 (20/80)
Weight (kg)	89 ± 21 (58 to 152)
Height (cm)	178 ± 12 (156 to 198)
BMI (kg/m^2^)	28 ± 5 (19 to 42)
Race, n (%)	
White	24 (96)
Native Hawaiian or other Pacific Islander	1 (4)
COVID-19, n (%)	20 (80)
Indication for therapeutic dose nadroparin, n (%)	
DVT	4 (16)
PE	13 (52)
SPAF	8 (32)
Serum creatinine (μmol/L)	97 ± 63 (40 to 283)
eGFR (ml/min/1.73 m^2^)	79 ± 29 (16 to 113)
CCr (mL/min)	105 ± 51 (15 to 201)
CCr ≥ 50 (n = 22)	115 ± 45 (51 to 201)
CCr ≥ 30 to 49 (n = 1)	44
CCr < 30 (n = 2)	20 ± 6 (15 to 24)
APACHE IV score	69 ± 18 (11 to 94)
SOFA score	4 ± 2 (0 to 8)
Daily dosage per bodyweight (IU/kg/d)	177 ± 34 (96 to 217)
CCr ≥ 50 (n = 22)	180 ± 31 (96 to 217)
CCr ≥ 30 to 49 (n = 1)	190
CCr < 30 (n = 2)	143 ± 63 (98 to 188)
Daily dosage per LBW (IU/kg/d)	254 ± 58 (144 to 401)
CCr ≥ 50 (n = 22)	257 ± 57 (144 to 401)
CCr ≥ 30 to 49 (n = 1)	297
CCr < 30 (n = 2)	200 ± 64 (155 to 245)
Daily dosage per IBW (IU/kg/d)	218 ± 54 (104 to 362)
CCr ≥ 50 (n = 22)	224 ± 53 (104 to 362)
CCr ≥ 30 to 49 (n = 1)	217
CCr < 30 (n = 2)	160 ± 60 (117 to 202)
No. of (identical) administrations (n)	10 ± 5 (4 to 25)
Invasive mechanical ventilation, n (%)	18 (72)
Vasopressor, n (%)	5 (20)
CRP (mg/L)	63 ± 62 (1 to 219)
Antithrombin (%)	115 ± 19 (88 to 156)^a^
Heart failure, history of	
NYHA I	18 (72)
NYHA II	6 (24)
NYHA III	1 (4)
Fluid balance (mL)	−105 ± 965 (−1711 to 2169)
Site of injection, n (%)	
Thigh	13 (52)
Abdomen	11 (44)
Unknown	1 (4)

Values are expressed as the mean ± SD (range), unless stated otherwise. Twice-daily 86-IE/kg nadroparin was considered as the unadjusted standard dosage (100%). IBW_female_ = 45.5 kg + 0.91 kg × (height in centimeters − 152.4); IBW_male_ = 50 kg + 0.91 kg × (height in centimeters −152.4). LBW_female_= (9270 × weight in kilograms)/(8780 + [244 × BMI]); LBW_male_ = (9270 × weight in kilograms)/(6680 + [216 × BMI]).

APACHE IV, Acute Physiology and Chronic Health Evaluation; BMI, body mass index; CCr, creatinine clearance (urine creatinine); CRP, C-reactive protein; DVT, deep vein thrombosis; eGFR, estimated glomerular filtration rate (Chronic Kidney Disease Epidemiology Collaboration in mL/min/1.73 m^2^); F, female; IBW, ideal body weight; LBW, lean body weight; M, male; NYHA, New York Heart Association; SOFA, Sequential Organ Failure Assessment; SPAF, stroke prevention in atrial fibrillation; PE, pulmonary embolism.

a One missing data point, n = 24.

In the 3- to 5-hour time interval, the lowest observed anti-Xa activity was 80% and the highest observed activity was equal to the peak anti-Xa activity (100%). Thus, in all patients, the measured anti-Xa activities were representative for the actual peak anti-Xa activity. Therefore, conditions were met to regard the measurement of an anti-Xa activity in the 3- to 5-hour time interval as an acceptable method to measure the peak anti-Xa activity (*p* < .023).

**Figure 2:**
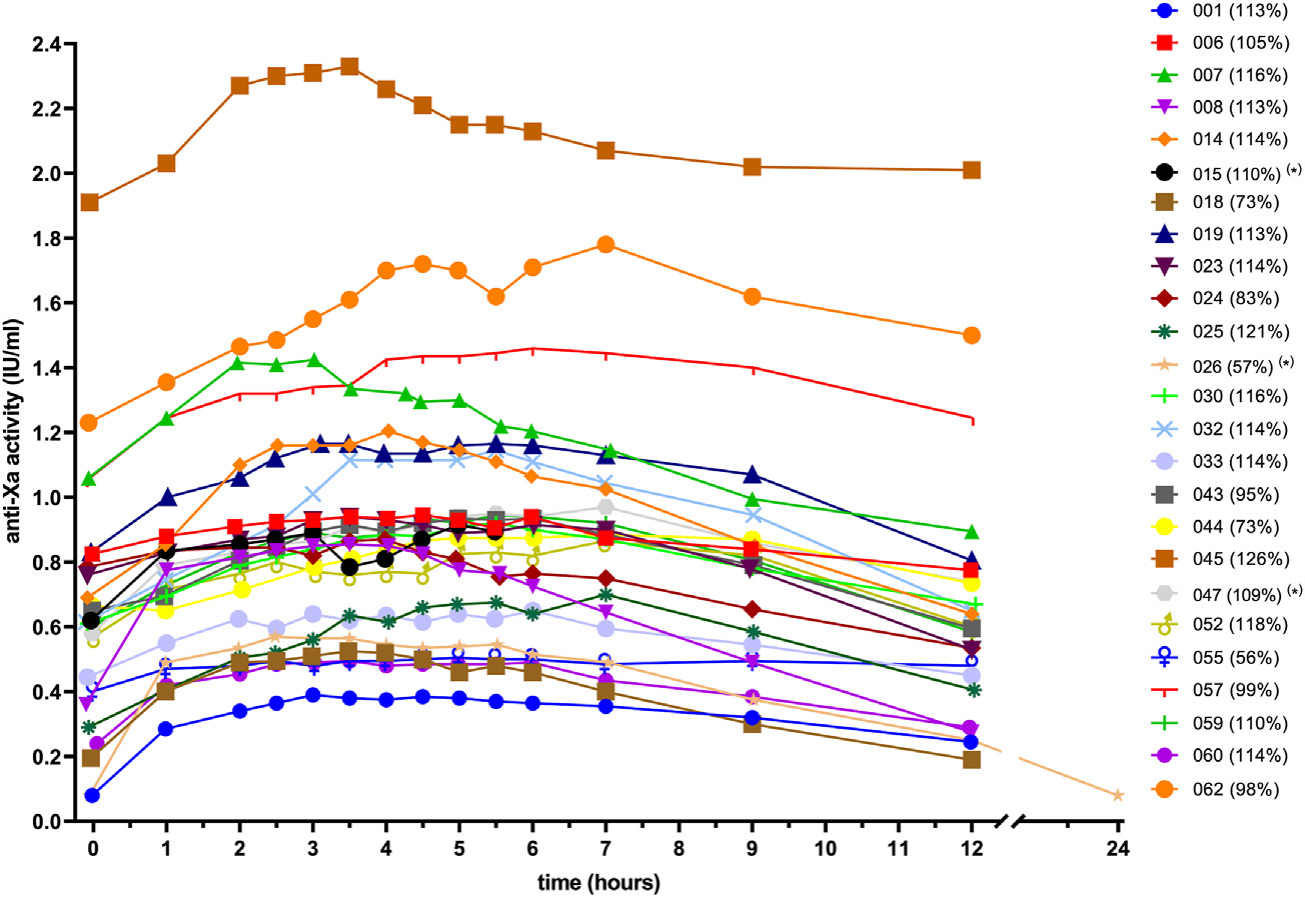
Per-patient plasma anti-Xa activity vs. time curves of a subcutaneously administered therapeutic nadroparin dose in critically ill patients (n = 25). The legend shows per patient the study number and the percentage (%) of a “standard daily dosage” of nadroparin as received by each patient. Twice-daily 86 IE nadroparin per kilogram bodyweight was considered a “standard daily dosage” (100%). ∗Patients with renal failure (015: creatinine clearance [CCr], 44 mL/min; 026: CCr, 15 mL/min; 047: CCr, 24 mL/min). Patients 044 and 055 had a bodyweight of >110 kg. In patient 026, plasma anti-Xa activity was below the limit of blank (<0.15 IU/mL) at t = 0 and t = 24 hours.

Two patients with a bodyweight of >110 kg (119 and 152 kg) were included after a prior dose reduction and change from Fraxiparine forte (nadroparin 19,000 IE/mL concentration) to Fraxiparine (nadroparin 9500 IE/mL concentration). Among the 25 patients who were evaluated, 24 patients received nadroparin twice daily. One patient was treated once daily with nadroparin, this patient had a urine CCr of <30 mL/min. One other patient was lost to follow-up with regard to secondary objectives because of the need of acute surgery. Renal function of the patients was predominantly good. One participant showed a CCr between 30 and 50 mL/min, and 2 patients were suffering from severe renal failure (CCr, <30 mL/min). In 1 patient, the through plasma anti-Xa activity was below the LOB. The per-patient plasma anti-Xa activity vs time curves are shown in Figure 2, and the mean plasma anti-Xa activity vs. time curve is shown in the Supplementary Figure.

Details of the nadroparin PK parameters are shown in Table 3. The t_max_ was observed between 2.5 and 7.0 hours after injection of nadroparin, with a median t_max_ of 5 hours. The median apparent elimination half-life was 9.6 hours. The mean apparent elimination half-life of nadroparin was 18.1 hours. The right-skewed distribution of apparent half-lives was caused by 2 remarkable long elimination half-lives (65 and 121 hours). Both these long apparent elimination half-lives were measured in participants with adequate renal function (CCr 114 and 73 ml/min). One of the shortest apparent elimination half-lives (6.8 hours) and a trough activity below LOB was determined in one of the 2 patients with severe renal clearance (CCr, 15 mL/min).

**Table 3:** Pharmacokinetic parameters of therapeutic nadroparin in critically ill patients.

Parameters	All patients	CCr ≥ 50 (mL/min)	CCr ≥ 30-49 (mL/min)	CCr < 30 (mL/min)
Primary endpoint				
Patients (n)	25	22	1	2
Lowest anti-Xa (3-5 h) relative to C_max_ (%)	93 ± 5 (80-100)	93 ± 5 (80-100)	97	92 ± 4 (89-95)
Underestimated peak anti-Xa activities 3 h and 5 h after injection,^a^ n (%)	0 (100)	0 (88)	0 (4)	0 (8)
Secondary endpoints				
Patients (n)		22	1	2^b^
C_max_ (IU/mL)	0.98 ± 0.43 (0.39-2.33)	1.00 ± 0.45 (0.39-2.33)	0.92	0.77 ± 0.28 (0.57-0.97)
C_min_ (IU/mL)	0.66 ± 0.43 (0.08-2.01)	0.69 ± 0.43 (0.19-2.01)	c	0.41 ± 0.47 (0.08-0.74)
t_max_ (h)	5.00 (2.50-7.00)	5.25 (3.50-7.00)	5.00	5.25 (3.00-7.00)
AUC (0-12 h) (h·IU/mL)	10.3 ± 4.8 (3.9-25.3)	10.3 ± 5.0 (3.9-25.3)	c	10.3^b^
AUC (0-24 h) (h·IU/mL)	7.3			7.3^b^
k_a_ (h-1)	0.216 ± 0.218 (0.037-0.956)	0.186 ± 0.165 (0.037-0.720)	0.161	0.569 ± 0.547 (0.182-0.956)
t½ absorption (h)	5.9 ± 4.3 (0.7-18.8)	6.3 ± 4.4 (1.0-18.8)	4.3	2.3 ± 2.2 (0.7-3.8)
k_e_ (h-1)	0.072 ± 0.040 (0.006-0.172)	0.071 ± 0.041 (0.006-0.172)	c	0.078 ± 0.033 (0.054-0.101)
t½ elimination (h)	18.1 ± 25.1 (4.0-120.9)	18.8 ± 26.2 (4.0-120.9)	c	9.8 ± 4.2 (6.8-12.8)

Data are presented as arithmetic mean ± SD (range), except for values of t_max_, which are presented as median (range).

AUC, area under the anti-Xa activity vs time curve from injection, 0 to 12 hours or 24 hours; CCr, creatinine clearance (urine creatinine); C_max_, the actual maximal peak anti-Xa activity; C_min_, trough plasma activity; k_a_, the apparent absorption rate constant; k_e_, the apparent elimination rate constant; t_½_ absorption, the apparent absorption half-life; t_½_ elimination, the apparent elimination half-life; t_max_, the time of actual peak anti-Xa activity (C_max_).

a Underestimated if ([lowest measured anti-Xa in interval 3 to 5 hours]/[C_max_])× 100% is <80%.

b CCr < 30 mL/min: AUC (0-24 hours) for patient with once-daily nadroparin (57% of standard dose) and AUC (0-12 hours) for patient with twice-daily nadroparin (109% of standard dose).

c Pharmacokinetic parameters from elimination phase are missing in 1 patient because of incomplete follow-up (acute surgery).

The individual PK curves could be visually divided into 3 groups. One group of participants showed a classical PK profile with a fast rise, distinct peak, and clear decline of anti-Xa activity. A second group of participants showed significant delayed elimination. In the third group of participants, anti-Xa activity vs time curves were remarkably flat. In this last group both the elimination and absorption half-lives were significantly prolonged. See the Supplementary Figure for 3 representative anti-Xa activity vs time curves.

**Figure 3:**
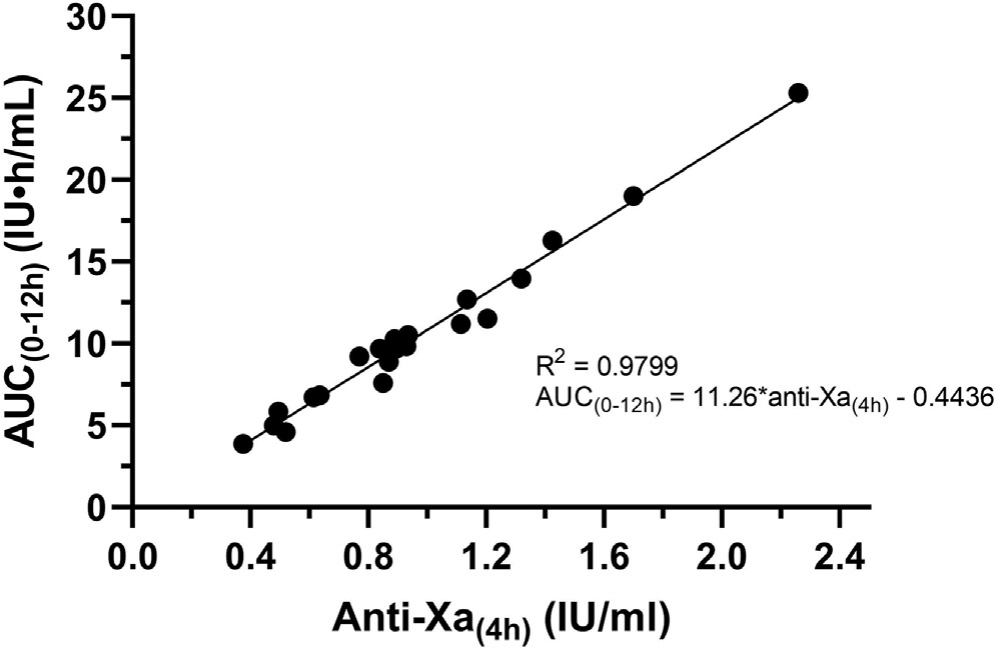
Aera under the curve _(AUC 0-12h)_ vs the anti-Xa activity 4 hours after injection (n = 23). One patient was lost to follow-up with regard to secondary objectives, and another patient was sampled for 24 hours (AUC_[0-24h]_).

Linear regression showed that the 4-hour anti-Xa activity activities were strongly correlated with the corresponding areas under the plasma anti-Xa activity vs. time curves (*R*^2^ = 0.9799; Figure 3).

We observed a large variation in total exposure to nadroparin between the participants. There was an up to 6-fold difference in the AUC_(0-12h)_ in the population with adequate renal function. The lowest nadroparin exposure (AUC_(0-24h)_, 7.3 IU·h/mL) was observed in a patient with (long lasting) severe renal insufficiency (CCr, 15 mL/min). This patient received 57% of the standard dose once daily. Another patient with severe renal insufficiency (CCr 24 mL/min) received an unadjusted full dose of nadroparin and showed a nadroparin exposure comparable to the mean AUC of all participants (AUC_(0-12h)_ 10.3 IU·h/mL). The mean C_max_ in the patients with adequate renal function was at the upper threshold of the general accepted therapeutic reference, 1.00 IU/mL (twice-daily dose of nadroparin).

## 4 Discussion

In this study, we found that measuring anti-Xa activity in a 3- to 5-hour interval after subcutaneous administration of therapeutic nadroparin in ICU patients does not lead to underestimation of peak anti-Xa activity. All anti-Xa activities in this time interval were in the range of 80% to 100% of the actual peak anti-Xa activity. This finding confirms the recommendation that peak anti-Xa activity can be measured in this broad 3- to 5-hour interval [5].

We observed large variations in both the apparent rates of absorption (k_a_) and apparent rates of elimination (k_e_). As the peak concentration of a drug occurs when the rate of drug absorption is equal to the rate of drug elimination, these large variations introduced a variable t_max_ ranging from 2.5 to 7.0 hours. As concluded in our primary objective, this broad range did not lead to clinically relevant underestimations of the actual peak anti-Xa activity. The measured median t_max_ of 5 hours and the broad range (2.50-7.00 hours) after subcutaneous administration are consistent with findings by others [[9], [10], [11],^16^,^17^].

The mean apparent absorption half-life (5.9 hours) was much longer than expected from literature and showed a large variation from <1 hour to up to almost 19 hours. Also, the mean apparent elimination half-life of 18.1 hours was much longer than that reported for nadroparin, generally 3 to 4 hours, as measured after a single-dose subcutaneous injection [11,17,18]. Very interesting was the observation that most of the obtained anti-Xa activity vs time curves were either classical, remarkably flat, or a mix of both. In the group of patients with a flat anti-Xa activity curve, both the apparent absorption half-lives and apparent elimination half-lives were extremely prolonged, suggesting the rate of elimination being dependent on the rate of absorption (ie, flip-flop PKs) [19]. Interestingly in an early crossover study with nadroparin in healthy volunteers by Rostin et al. [17], it was shown that after subcutaneous bolus injection of nadroparin the elimination half-life of the anti-Xa activity was doubled (around 4 hours) compared to the elimination half-life (around 2 hours) after injecting the nadroparin intravenously, suggesting the absorption rate of nadroparin from the site of injection being the rate limiting step for drug clearance. It should be noted that we did not perform modeling with PK software, so we could not calculate the real k_a_ and real k_el_. As elimination starts directly from the time of administration of nadroparin, the observed k_a_ does not represent the real absorption constant. Also, during the elimination phase, some absorption is still present.

Compared to earlier studies which reported elimination half-lives ranging from 3 to 5 hours in patients with renal impairment, the mean apparent elimination half-life of 18.1 hours we found in patients with adequate renal function was much longer. In fact, this long apparent elimination half-life and the 6-fold variance in peak anti-Xa activity despite adequate renal function may reject renal impairment as a cause of accumulation of nadroparin, which was suggested in these studies [10,20,21]. We propose that the elimination half-life of nadroparin can be prolonged by a risk factor independent from renal clearance. Our data suggest a possible saturation in the absorption, decreasing the rate of elimination. As studies with enoxaparin and dalteparin also have suggested renal saturation as concluded on high peak anti-Xa activities and prolonged elimination half-lives in renal impairment, it would be interesting to investigate if a comparable phenomenon is also observed in these LMWHs [10,[22], [23], [24]].

In patients with ultralong elimination half-lives (in our study up to 121 hours), care should be taken with planning surgical interventions or other procedures with a high bleeding risk. Furthermore, in these patients, steady state is not realized after 4 subsequent administrations of nadroparin. Therefore, in such patients carefully planned measurement of the anti-Xa activity is needed to guide therapy. Unfortunately, it is not possible to identify such patients as risk factors for prolonged elimination half-lives of subcutaneously administered nadroparin are yet unknown.

The 4-hour anti-Xa activity correlated strongly with the AUC_(0-12h)_ (*R*^2^ = 0.9799) and can therefore be considered to be a very representative estimate of the total nadroparin exposure in ICU patients. Furthermore, we observed a more than 6-fold difference in the AUC_(0-12h)_ of participants with adequate renal function. Small differences in AUCs can partly be explained by the use of 1 general dose of nadroparin for a broader weight range (Table 1). As the bioavailability of nadroparin is generally described to be near 100%, we do not expect that incomplete absorption can account for such large variation in nadroparin exposure [16,17,25]. Considering that the volume of distribution of LMWHs is of the same order of magnitude as the plasma volume, possibly the bodyweight-based dosing and the plasma volume being nonlinear to this bodyweight contributed to this large variation in the AUC_(0-12h)_ [11,16,26].

A limitation of this study is that a thromboembolic event related to COVID-19 was the predominant indication for patients being treated with nadroparin. Therefore, patients with COVID-19 were overrepresented in the general ICU population. Furthermore, of 75 patients assessed for eligibility, 25 patients were evaluated. Although the ratio screened vs evaluated patients in our study is reasonably comparable to those in other studies, the number of patients (or substitute decision makers) who declined to participate is fairly high [27,28]. In line with Dutch Law, no information is available on patients who declined to participate. We think the intensive sampling schedule in combination with limited access to substitute decision makers for patients transferred to our ICU from outside our region due to COVID-19, hampered consent and may impact generalizability. Moreover, generalizability may be a common limitation of a study in critically ill patients such as ours. In general, the population of ICU patients consists of several subpopulations of patients (eg, trauma, sepsis, and after surgery, among others), so in clinical trials, heterogeneity of effect can be expected [29]. Therefore, our findings are applicable to patient populations resembling the patient population in our study and should be taken with care in populations deviating from ours. Additionally, we did not measure clinical outcome or bleeding complications related to the nadroparin exposure.

Besides these limitations, a strong point of this study is that it was conducted prospectively. Patients were treated according to a standardized protocol, and anti-Xa samples were collected according to a fixed sampling scheme. Also, we were able to include many patients who received therapeutic nadroparin for a longer time before inclusion, which is different from other PK studies with nadroparin. With 14 sample points in 12 hours, we obtained the anti-Xa activity vs time curves very accurately, including the 12-hour exposure to therapeutic nadroparin. We estimated renal function precisely by collecting the 24-hour urine on study day to measure CCr. As far as we know, this is the first study to show significant prolongation of the elimination half-life of a LMWH despite adequate renal function.

## 5 Conclusion

Based on our data, we conclude that measuring anti-Xa activity 3 to 5 hours after therapeutic nadroparin administration provides an adequate estimate of the actual anti-Xa peak activity. The 4-hour anti-Xa activities correlated strongly with the total drug exposure to nadroparin. The general dosing scheme led to a 6-fold variation in drug exposure and a delayed elimination despite adequate renal function.
