# Supplementary material for: The right time to measure anti-Xa activity in critical illness: pharmacokinetics of therapeutic dose nadroparin
Source: Res Pract Thromb Haemost. 2023 May 20;7(4):100185. doi: 10.1016/j.rpth.2023.100185 (PMC10336191; doi:10.1016/j.rpth.2023.100185)
Supplement: Appendix [file mmc1.pdf]

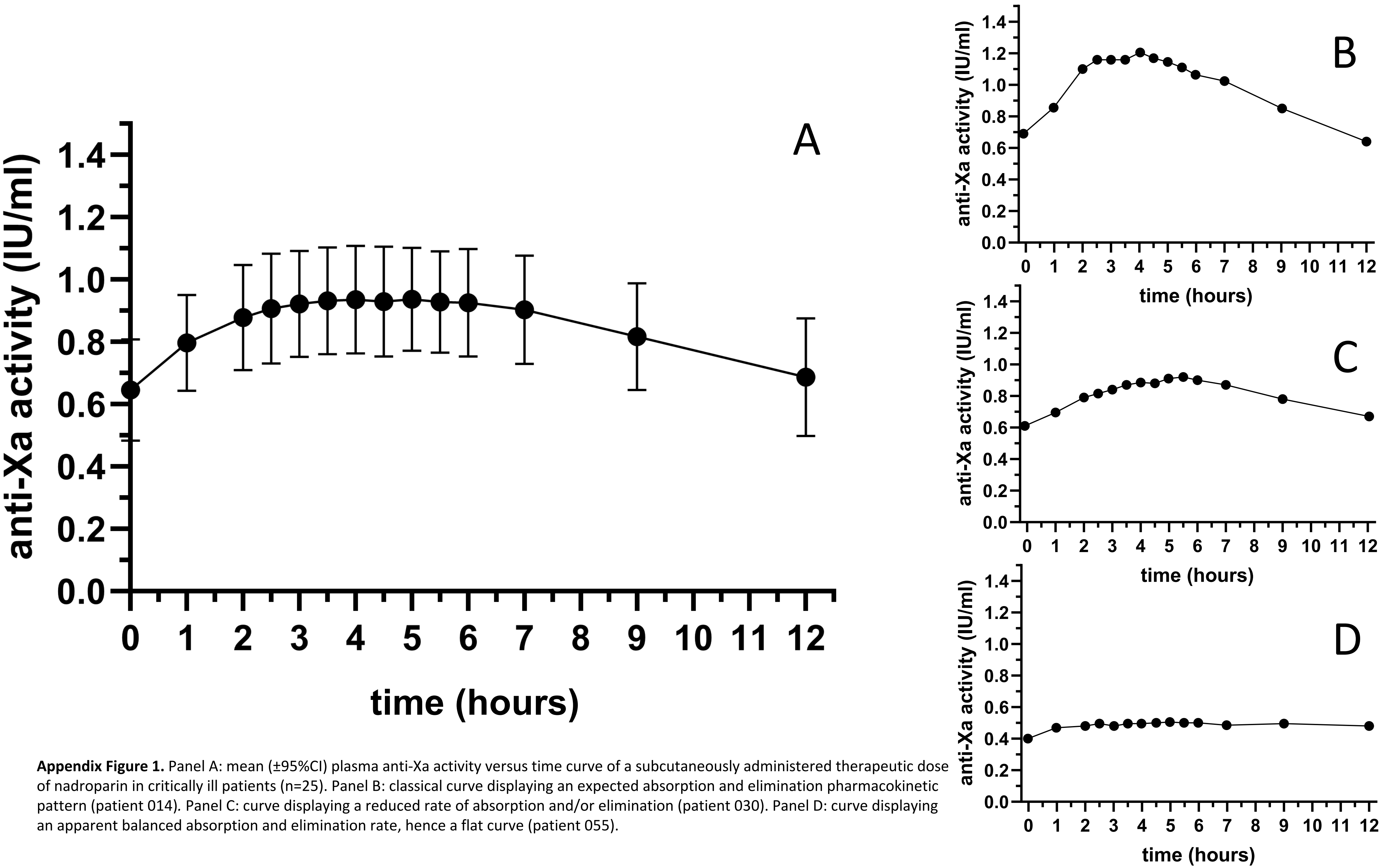

Appendix Figure 1. Panel A: mean (±95%CI) plasma anti-Xa activity versus time curve of a subcutaneously administered therapeutic dose of nadroparin in critically ill patients (n=25). Panel B: classical curve displaying an expected absorption and elimination pharmacokinetic pattern (patient 014). Panel C: curve displaying a reduced rate of absorption and/or elimination (patient 030). Panel D: curve displaying an apparent balanced absorption and elimination rate, hence a flat curve (patient 055).
